# Long-Term Hyperglycemia Causes Depressive Behaviors in Mice with Hypoactive Glutamatergic Activity in the Medial Prefrontal Cortex, Which Is Not Reversed by Insulin Treatment

**DOI:** 10.3390/cells11244012

**Published:** 2022-12-12

**Authors:** Ji Hyeong Baek, Hyeonwi Son, Jae Soon Kang, Dae Young Yoo, Hye Jin Chung, Dong Kun Lee, Hyun Joon Kim

**Affiliations:** 1 Department of Anatomy and Convergence Medical Sciences, Institute of Health Sciences, Tyrosine Peptide Multiuse Research Group, Anti-Aging Bio Cell Factory Regional Leading Research Center, Gyeongsang National University Medical School, 15 Jinju-daero 816 Beongil, Jinju 52727, Republic of Korea; 2 College of Pharmacy and Research Institute of Pharmaceutical Sciences, Gyeongsang National University, 501 Jinju-daero, Jinju 52828, Republic of Korea; 3 Department of Physiology, Institute of Health Sciences, Gyeongsang National University Medical School, 15 Jinju-daero 816 Beongil, Jinju 52727, Republic of Korea

**Keywords:** hyperglycemia, depression, glutamatergic neurotransmission, prefrontal cortex, insulin receptor signaling

## Abstract

The etiology of hyperglycemic-induced depressive behaviors is unclear. We hypothesized that long-term hyperglycemia may induce long-lasting disturbances in glutamatergic signaling and neural damages, causing depressive behaviors. To prove our hypothesis, a C57BL/6N mouse model of hyperglycemia was maintained for 4 weeks (equivalent to approximately 3 years in humans), after which insulin treatment was administered for an additional 4 weeks to normalize hyperglycemia-induced changes. Hyperglycemic mice showed depressive-like behaviors. Glutamatergic neurons and glial cells in the medial prefrontal cortex (mPFC) were affected by hyperglycemia. Insulin treatment improved blood glucose, water intake, and food intake to normoglycemic levels, but did not improve depressive-like behaviors. Glutamatergic signaling decreased with long-term hyperglycemia and did not normalize with insulin-induced normoglycemia. Importantly, hyperglycemia-induced changes in the mPFC were almost not reversed by the 4-week insulin treatment. In particular, levels of insulin receptor beta subunit (IRβ), IRS-1, vesicular glutamate transporter 1, glutamine transporter SNAT2, phosphate-activated glutaminase, and GLUT-3 were not changed by insulin. Nitration and the dephosphorylation of IRβ in the PFC also did not improve with insulin treatment. Therefore, our results suggest that hypoactive glutamatergic activity in the mPFC is involved in diabetic-associated depressive behaviors, and it is difficult to cure with glycemic regulation alone.

## 1. Introduction

Major depressive disorder is a devastating psychiatric illness that reduces a patient’s quality of life and sometimes leads to suicide. The prevalence of depression is two to three times higher in people with diabetes (20–30%) than in those without diabetes (5–15%) [1,2,3]. With the rising prevalence of diabetes worldwide, the prevalence of depression is expected to increase as well. However, the nature of the relationship between depression and diabetes remains unclear.

Because hyperglycemia is a major feature of diabetes, efforts have focused on controlling depression by optimizing glucose management using insulin or glycemia-control agents. However, whether insulin treatment improves depression in patients with diabetes remains controversial. Some studies have noted that insulin therapy improves symptoms of depression in patients with type 2 diabetes, whereas other studies have noted that it does not [4,5]. There have also been frequent reports of depressive symptoms in patients with type 1 diabetes despite continuous insulin treatment [2].

Hyperglycemia decreases cell proliferation, induces mitochondrial dysfunction, increases oxidative/nitrative stress (which leads to cellular biomolecular damage), impairs glutamate metabolism, and increases pro-inflammatory cytokine release [6,7]. It also directly affects intraneuronal glucose levels. As a result, the brain is particularly vulnerable to fluctuations in blood glucose levels [8]. Blood glucose fluctuations, especially from high glucose levels to glucose deprivation, also exacerbate glial injuries [6]. It has been suggested that these changes in the brain result in abnormal neurotransmission [6], ultimately causing depressive symptoms [2,6,9].

According to previous studies, it can be difficult to effectively control depressive symptoms by simply regulating blood glucose levels [2,4,5]. Therefore, to manage hyperglycemia-induced depressive behaviors, it is necessary to reverse the brain damage caused by hyperglycemia, in addition to controlling blood glucose levels. To accomplish this, it is essential to first characterize the neural damage that fails to recover with glycemic control alone, as this will yield the identification of targets for managing the depressive symptoms of patients with diabetes.

Therefore, we hypothesized that long-term continuous hyperglycemia leads to neural damage that is irreversible or difficult to resolve with insulin treatment alone. In the present study, we first identified depressive-behavior-associated alterations in the mouse brain resulting from streptozotocin (STZ)-induced hyperglycemia. We then investigated whether the behavioral and molecular changes secondary to hyperglycemia were reversed by insulin treatment in an attempt to identify potential novel therapeutic targets for regulating depressive symptoms in patients with diabetes.

## 2. Materials and Methods

### 2.1. Animals

Male 7-week-old C57BL/6N mice (20–23 g body weight; Koatech, Pyeongtaek, Republic of Korea) were habituated for 1 week prior to the experiments in a specific-pathogen-free animal facility at the School of Medicine, Gyeongsang National University. The mice were housed individually to monitor their health statuses by measuring individual food intake and water consumption. Animals were given free access to laboratory chow and water under a 12 h light/dark cycle (lights on at 06:00 a.m.) at a constant temperature (25 ± 2 °C). For electrophysiologic experiments, we used VGLUT2-CRE::tdTomato or VGLUT2-CRE::EGFP mice developed in our previous study [10,11]. These animals express fluorescent proteins in cells expressing the VGLUT2 protein, which are predominantly glutamatergic neurons. Animal use procedures were performed in accordance with the National Institutes of Health (NIH) guidelines and a protocol (GNU-180619-M0029) approved by the Gyeongsang National University Institutional Animal Care and Use Committee.

### 2.2. STZ-Induced Hyperglycemia Model and Insulin Treatment

Eight-week-old mice were randomly assigned to one of two groups (CTL, control mice; STZ, STZ-injected mice) using a computer-generated list according to body weight. The mice were fasted overnight and intraperitoneally injected with a 50 mM sodium citrate buffer (pH 4.5) or STZ (Sigma-Aldrich, St. Louis, MO, USA) in a sodium citrate buffer, respectively. A total of 100 mg STZ/kg body weight was given to C57BL/6N and VGLUT2-CRE::EGFP animals, and 150 mg/kg was given to VGLUT2-CRE::tdTomato animals. STZ doses were basically determined according to the guide of a previous study [12]. STZ doses for the transgenic strains (VGLUT2-CRE::tdTomato and VGLUT2-CRE::EGFP) were determined by the amount in which the blood glucose level of 80% of mice rose to 400 mg/dL or higher within 1 week after the STZ injection. Blood glucose levels obtained from tail blood were measured every week using a glucometer (FreeStyle Optium Neo, Abbott, Witney, UK). After 3 weeks, behavioral tests were conducted for 1 week to evaluate depressive behaviors and social interaction behaviors. STZ mice were then divided into STZ and STZ + insulin (SI) groups and implanted with blank pellets or insulin pellets (LinBit, Linshin Canada, Scarborough, ON, Canada), respectively, according to the manufacturer’s instruction (2 pellets per animal in this study). Blood glucose levels were measured twice per week. After 3 weeks, the presence of anhedonic behaviors was evaluated. Insulin was administered for a total of 4 weeks before tissue sampling. Brain tissues were collected and immediately frozen in liquid nitrogen.

### 2.3. Behavioral Assessments

The Y-maze test was performed to evaluate exploratory behavior and memory, as described in a previous study [13]. Social interaction tests were also performed as described previously [11]. The sucrose preference test (SPT), tail suspension test (TST), and female urine sniffing test (FUST) were performed to identify symptoms of anhedonia and despair, as previously described [13,14]. Animal behavior was recorded and analyzed using EthoVision XT7 (Noldus, Wageningen, The Netherlands).

### 2.4. Immunohistochemistry

Two mice in each group were deeply anesthetized with avertin and perfused with PBS (pH 7.4) and 4% (wt/vol) paraformaldehyde in PBS. Brains were collected, post-fixed, and sectioned at 40 μm thickness using a vibratome (Leica VT1200, Leica Biosystems, Nussloch, Germany). Brain sections were incubated at 4 °C overnight with antibodies for ΔFOSB (1:200, 2251S, Cell Signaling; stains, both FOSB and ΔFOSB but mainly ΔFOSB, because FOSB degrades with time, leaving ΔFOSB), glial fibrillary acidic protein (GFAP; 1:200, Z0334, Dako, Glostrup, Denmark), glutamine synthetase (GS; 1:200, MAB302, Millipore, Temecula, CA, USA), ionized-calcium-binding adaptor molecule 1 (IBA1; 1:200, ab5076, Abcam, Cambridge, UK), sodium-dependent neutral amino acid transporter 2 (SNAT2; 1:20, sc-166366, Santa Cruz, Dallas, TX, USA), phosphate-activated glutaminase (PAG; 1:200, AB113509, Abcam), insulin receptor β (IRβ; 1:20, sc-57342, Santa Cruz), insulin receptor substrate-1 (IRS-1; 1:200, 06-248, Millipore), vesicular glutamate transporter 1 (VGLUT1, 1:20, 48-2400, Invitrogen, Carlsbad, CA, USA), and VGLUT2 (1:20, 42-7800, Invitrogen). The slices were then incubated with Alexa Fluor 594- and/or 488-conjugated secondary antibodies (1:200, Invitrogen). Digital images were captured using a spinning-disk confocal microscope (Olympus, Tokyo, Japan) and analyzed with ImageJ software (NIH).

### 2.5. Western Blot Analysis

Tissues were lysed with glass beads in an RIPA buffer (Elpis-Biotech, Daejeon, Republic of Korea) using a Bullet Blender (Next Advance, New York, NY, USA), followed by sonication for 2 min and centrifugation at 12,000× *g*, 4 °C for 10 min. Protein samples (10 μg) were separated by SDS-PAGE and transferred onto PVDF membranes. The membranes were incubated at 4 °C overnight with a primary antibody (1:200–1:1000), and then incubated with a secondary antibody conjugated to horseradish peroxidase (1:10,000). Immunoblot signals were detected using an enhanced chemiluminescence detection kit (Thermo Fisher Scientific, Waltham, MA, USA) and iBright FL1000 (Thermo Fisher Scientific). Band intensities were determined using iBright analysis software.

### 2.6. Immunoprecipitation

Protein samples (400 μg, pooled by groups) were incubated with a 4 μg anti-IRβ antibody (07-724; Millipore) at 4 °C for 3 h. Protein A/G-plus agarose (sc-2003; Santa Cruz; 30 μL) was added, and the samples were incubated at 4 °C overnight. IRβ was eluted with an SDS sample buffer and used for Western blot analysis of nitrotyrosine (1:200, 06-284, Millipore) and phospho-IRβ (Y1345) (1:200, 3026, Cell Signaling).

### 2.7. Measurements of GS Activity, ROS/RNS, and Amino Acid Levels

A GS activity assay was conducted as described in a previous study [13,14]. The total reactive oxygen/reactive nitrogen species (ROS/RNS) levels in plasma and tissue samples were measured using an OxiSelect ROS/RNS assay kit (STA-347; Cell Biolabs, San Diego, CA, USA). Levels of glutamate (Glu), glutamine (Gln), and γ-aminobutyric acid (GABA) in the plasma and prefrontal cortex (PFC) were quantified as previously described [13,14].

### 2.8. Glutamatergic Neurotransmission Activity Analysis

Transverse-sectioned brain slices (200 μm thickness) were prepared as previously described [10,15]. Whole-cell voltage-clamp recordings were obtained from visualized glutamatergic neurons in the medial PFC (mPFC) at a holding potential of −70 mV. The glutamatergic current was isolated using picrotoxin (100 μM). Membrane currents were recorded with a multi-clamp 700B in whole-cell configuration. All recordings were made at 30 ± 2 °C. The slices were continuously superfused with an oxygenated artificial cerebrospinal fluid, including 10 mM glucose at a 1.5 mL/min flow rate. The pipette solution contained the following substances: 130 mM KCl; 5 mM CaCl_2_; 10 mM EGTA; 10 mM HEPES; 2 mM MgATP; 0.5 mM Na_2_GTP; and 5 mM phosphocreatine.

### 2.9. Statistical Analysis

All data are presented as mean ± SEM. Statistical significance was determined using a two-tailed unpaired Student’s t-test, one-way ANOVA with a Newman–Keuls post-hoc test, and a two-way ANOVA with Tukey’s post-hoc test. Details of the statistical tests used are provided in the figure legends. Data summaries and statistical analyses were performed using GraphPad Prism 7 (GraphPad Software, La Jolla, CA, USA). Statistical significance was set at *p* < 0.05.

## 3. Results

### 3.1. Hyperglycemia Was Induced by STZ Injection

**Figure 1:**
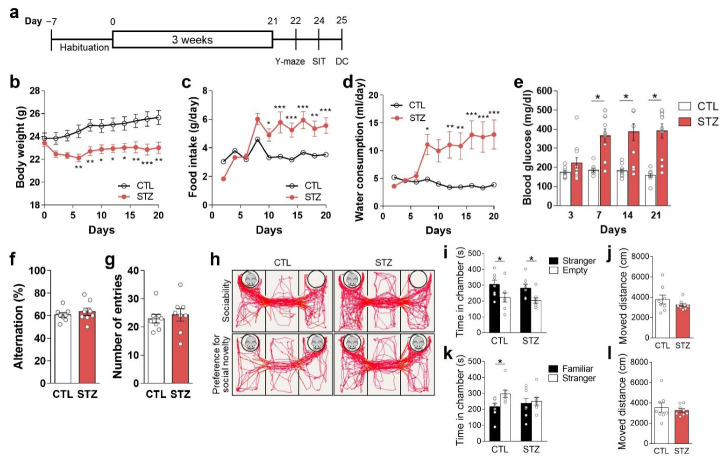
Streptozotocin (STZ)-induced hyperglycemia caused anhedonic behaviors. (**a**–**e**) Construction of an STZ-induced hyperglycemia model: (**a**) experimental design and changes in (**b**) body weight; (**c**) food intake; (**d**) water consumption; and (**e**) blood glucose levels of control (CTL) and STZ-injected (STZ) mice over 3 weeks. (**f**,**g**) Y-maze results: (**f**) spontaneous alternations and (**g**) total number of entries into the 3 arms of the Y-maze apparatus. (**h**–**l**) Hyperglycemia-induced anhedonic behaviors: (**h**) representative movement-tracking images for social interaction tests, chamber exploration time, and moved distance in the sociability test (**i**,**j**), and the preference for social novelty test (**k**,**l**). Data are presented as mean ± SEM. (**b**–**d**) * *p* < 0.05, ** *p* < 0.01, *** *p* < 0.001 compared with the CTL group according to two-way ANOVA with Tukey’s post-hoc tests. (**e**,**i**,**k**) * *p* < 0.05 between indicated groups according to Student’s *t*-test. *n* = 8/group. DC, decapitation; SIT, social interaction test.

To induce hyperglycemia, a core feature of diabetes, we injected 8-week-old male mice with one dose of STZ (100 mg/kg, i.p.) (Figure 1a). The average blood glucose level of the STZ group was higher than 390 mg/dL (non-fasting condition) 3 weeks after STZ administration (Figure 1e). The STZ group exhibited other manifestations of diabetes, including reduced body weight and increased food intake and water consumption (Figure 1b–d).

### 3.2. Hyperglycemia Caused Anhedonic and Despair Behaviors

**Figure 2:**
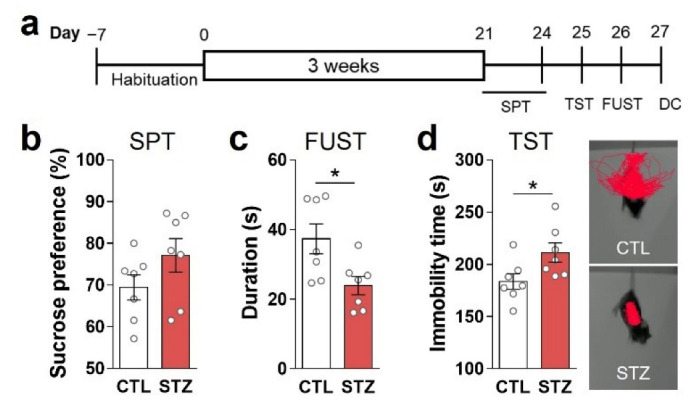
Hyperglycemia caused anhedonic and despair behaviors. (**a**) Experimental design. (**b**) Sucrose preference test (SPT). (**c**) Female urine sniffing test (FUST). (**d**) Tail suspension test (TST): immobility time and representative movement-tracking images. Data are presented as mean ± SEM. * *p* < 0.05 between indicated groups according to Student’s *t*-test. *n* = 8/group.

To investigate the effects of hyperglycemia on depressive-like behavioral changes, we evaluated social interactive, anhedonic, and despair behaviors (Figure 1 and Figure 2). We found that the preference for novel social interaction was reduced in STZ mice (Figure 1h,k,l), but not the basal preference for social interaction (Figure 1h–j). These findings could represent the presence of anhedonic behaviors. The Y-maze results showed no differences between the CTL and STZ groups (Figure 1f,g), indicating that the difference in preference for novel social interaction between the two groups was not due to differences in cognitive function but was the result of actual preference differences.

To confirm the presence of anhedonic symptoms, we performed a SPT and FUST. The sniffing time of the STZ group for female urine was lower than that of the CTL group (Figure 2c), while sucrose preference was similar between the groups (Figure 2b). The STZ group also exhibited greater immobility during TST than the CTL group (Figure 2d).

### 3.3. Hyperglycemia-Induced Gliosis and Oxidative/Nitrative Stress in the Medial Prefrontal Cortex and Plasma

**Figure 3:**
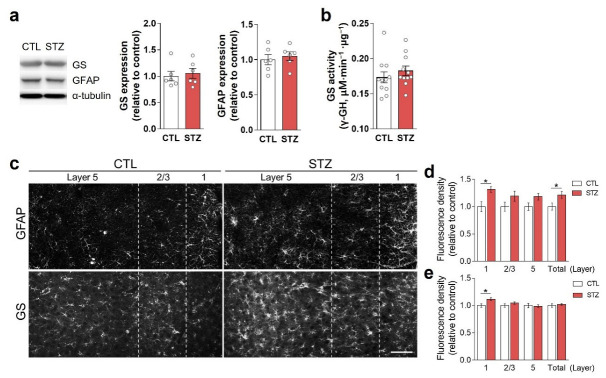
Hyperglycemia-induced astrocytic alterations in the medial prefrontal cortex (mPFC). (**a**) Western blot analysis of glutamine synthetase (GS) and glial fibrillary acidic protein (GFAP) using PFC crude lysates. The left panel shows representative immunoblot images, and the right panel shows quantifications. (**b**) GS activity in the PFC crude lysates. (**c**–**e**) Immunohistochemical analysis of GFAP (**c**,**d**) and GS (**c**,**e**) in the infralimbic mPFC. Scale bar = 50 μm. Data are presented as mean ± SEM. * *p* < 0.05 between indicated groups according to Student’s *t*-test. *n* = 6/group.

Immunoblotting using crude tissue lysates did not show changes in the expression of GS or GFAP in the STZ group compared to the CTL group (Figure 3a). GS activity was also unchanged by hyperglycemia (Figure 3b). However, in immunohistochemical analysis, GFAP and GS expression levels were increased in layer 1 of the mPFC in the STZ group (Figure 3c–e).

**Figure 4:**
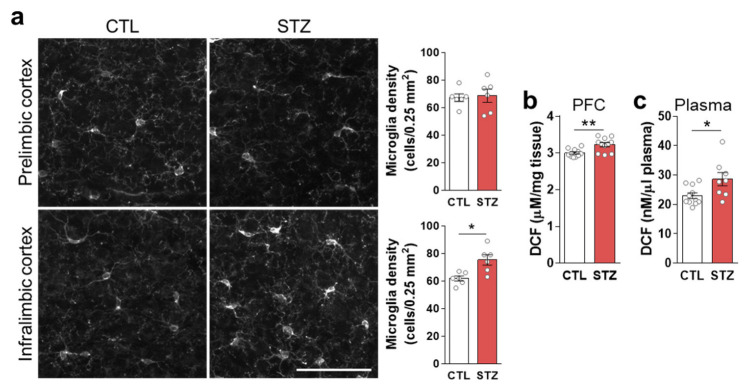
Hyperglycemia-induced gliosis and oxidative/nitrative stress. (**a**) Immunohistochemical analysis of microglia marked by anti-IBA1 (ionized-calcium-binding adaptor molecule 1) antibody in the prelimbic and infralimbic mPFC. The left panel shows representative images, and the right panel shows quantifications. Scale bar = 50 μm. (**b**,**c**) Reactive oxygen/nitrogen species analysis in the PFC (**b**) and plasma (**c**). Data are presented as mean ± SEM. * *p* < 0.05, ** *p* < 0.01 between indicated groups according to Student’s *t*-test. *n* = 6/group.

Microglia, marked by IBA1, were significantly increased in the infralimbic (IL) mPFC of the STZ group mice (Figure 4a). ROS/RNS levels were also increased in the PFC, as well as in the plasma, by hyperglycemia (Figure 4b,c).

### 3.4. Hyperglycemia Decreased Glutamatergic Neuronal Activity

**Figure 5:**
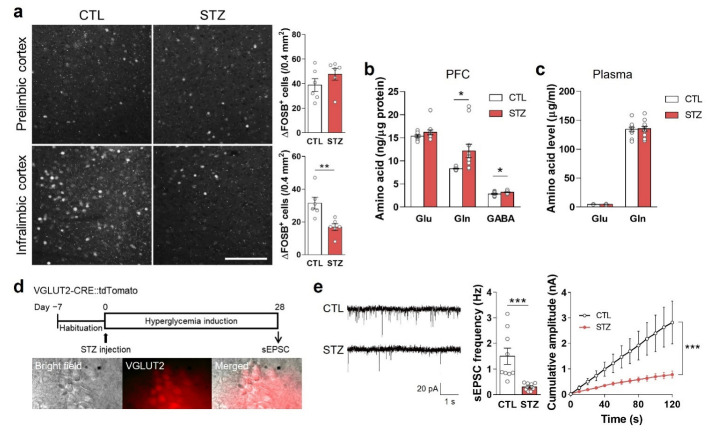
Hyperglycemia decreased glutamatergic neuronal activity. (**a**) Immunohistochemical analysis of ΔFOSB in the prelimbic and infralimbic mPFC. The left panel shows representative images, and the right panel shows quantifications. Scale bar = 100 μm. (**b**,**c**) Glutamate (Glu), glutamine (Gln), and γ-aminobutyric acid (GABA) levels in the PFC (**b**) and plasma (**c**) analyzed by LC-MS/MS. (**d**) Experimental design for electrophysiologic analysis and microscope images of spontaneous excitatory postsynaptic current (sEPSC) recording. sEPSC in the mPFC was measured 4 weeks after STZ injection in VGLUT2-CRE::tdTomato mice. (**e**) sEPSC and cumulative amplitude in glutamatergic neurons in the MPFC. *n* = 10/group (10 cells from 3 mice). Data are presented as mean ± SEM. * *p* < 0.05, ** *p* < 0.01, *** *p* < 0.001 between indicated groups according to Student’s *t*-test or two-way ANOVA with Tukey’s post-hoc tests.

In the STZ group, ΔfosB (which accumulates in response to repeated neural activation) was reduced in the IL mPFC but not in the prelimbic (PL) mPFC (Figure 5a). When evaluating Glu, Gln, and GABA levels in the PFC and plasma, Gln in the PFC was significantly increased in the STZ group (Figure 5b,c).

To measure the spontaneous excitatory postsynaptic current (sEPSC) during hyperglycemia, we prepared hyperglycemic VGLUT2-CRE::tdTomato mice using STZ injections. The frequency and amplitude of the sEPSC in this STZ group were significantly decreased compared to the CTL group (Figure 5d,e).

### 3.5. Insulin Treatment Partially Reversed Hyperglycemic Phenotypes

**Figure 6:**
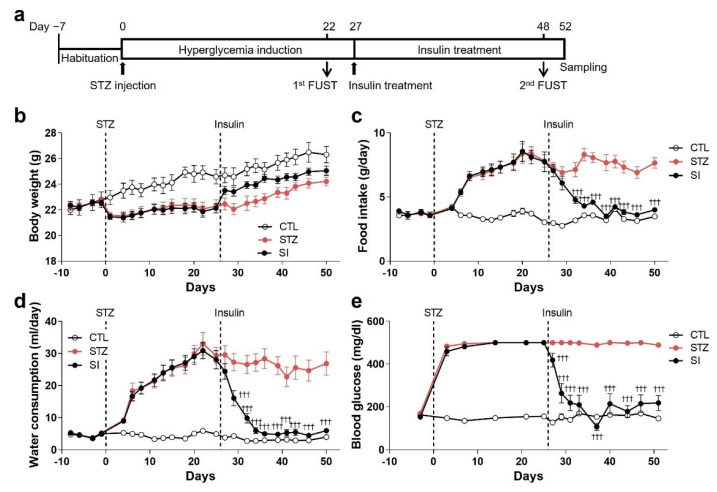
Insulin treatment reversed hyperglycemic phenotypes. (**a**) Experimental design to investigate the effects of insulin on hyperglycemia-induced alterations. After 4 weeks of hyperglycemia induction, insulin pellets were implanted and maintained for 4 weeks. (**b**–**e**) Changes in body weight (**b**), food intake (**c**), water consumption (**d**), and blood glucose levels (**e**). CTL, *n* = 7; STZ, *n* = 7; SI, insulin-treated STZ group, *n* = 11. Data are presented as mean ± SEM. ^†††^ *p* < 0.001 compared with the STZ group according to two-way ANOVA with Tukey’s post-hoc tests.

To investigate whether insulin treatment reverses phenotypic alterations induced by hyperglycemia, half of the animals in the STZ group were treated for 4 weeks with subcutaneously implanted insulin pellets beginning at the end of the 4th week of hyperglycemia (Figure 6a). Food intake, water consumption, and blood glucose levels were restored to those seen in normoglycemic animals with insulin treatment (Figure 6c–e). The body weight of the SI group mice also increased more than that of the STZ group (Figure 6b). These changes were evident from the 3rd day of insulin treatment and were stably maintained for 4 weeks.

### 3.6. Insulin Treatment Could Not Completely Reverse Hyperglycemia-Induced Depressive-like Behaviors and Alterations in the PFC

**Figure 7:**
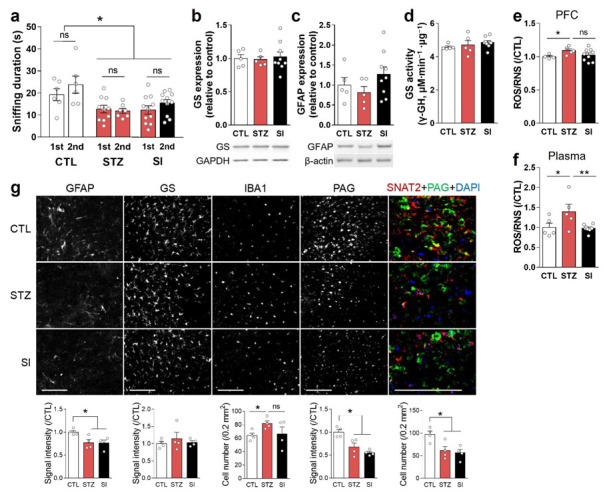
Insulin treatment could not completely reverse hyperglycemia-induced behavioral and alterations in the PFC. (**a**) Sniffing time in female urine sniffing tests conducted 3 weeks after STZ injection (1st) and 3 weeks after insulin implantation (2nd). CTL, *n* = 7; STZ, *n* = 7; SI, *n* = 11. (**b**,**c**) Representative images and quantification in Western blots of GS (**b**) and GFAP (**c**) using PFC crude lysates. (**d**) GS activity analysis using PFC crude lysates. (**e**,**f**) Reactive oxygen/nitrogen species (ROS/RNS) in the PFC (**e**) and plasma (**f**). (**b**–**f**) CTL, *n* = 5; STZ, *n* = 5; SI, *n* = 9. (**g**) Immunohistochemical analysis of GFAP, GS, IBA1, phosphate-activated glutaminase (PAG), and sodium-dependent neutral amino acid transporter 2 (SNAT2) in the infralimbic mPFC. The upper panel shows representative images, and the lower panel shows quantifications. *n* = 4/group. Scale bar = 100 μm. Data are presented as mean ± SEM. * *p* < 0.05, ** *p* < 0.01 between indicated groups according to one-way ANOVA with Newman–Keuls post-hoc tests.

To test the effects of insulin treatment on hyperglycemia-induced depressive behaviors, we performed the first FUST 3 weeks after the STZ injection. This test again confirmed the presence of hyperglycemia-induced anhedonic behaviors. A second FUST was performed 3 weeks after insulin treatment and revealed no significant difference in sniffing times between the STZ and SI group mice (Figure 7a). The STZ group, which had sustained hyperglycemia for 8 weeks, had reduced physical strength. Therefore, the behaviors of mice before and after insulin treatment were compared only with FUST, which required less tension and physical strength consumption.

In immunoblot analysis using crude PFC lysates, GFAP and GS expression levels were unchanged in both the STZ and SI groups, compared with the CTL group (Figure 7b,c). GS activity was also unchanged (Figure 7d). Region-specific immunohistochemistry analysis showed that GFAP, PAG, and SNAT2 were decreased in both the STZ and SI groups (Figure 7g). GFAP expression was increased at 4 weeks of hyperglycemia (Figure 3c,d) but decreased at 8 weeks, and did not increase with insulin treatment (Figure 7g).

There was no significant difference in ROS/RNS levels in the PFC between the STZ and SI groups (Figure 7e). Although IBA1 in the mPFC was reduced with insulin treatment, the difference between the STZ and SI groups was also not statistically significant (Figure 7g). By contrast, plasma ROS/RNS levels returned to normal with insulin treatment (Figure 7f). These results suggest that the mechanism(s) used to cope with glycemic changes differ between the mPFC and peripheral tissues.

### 3.7. Neurotransmitter and Hypoactive Glutamatergic Signaling Did Not Change with Insulin Treatment

**Figure 8:**
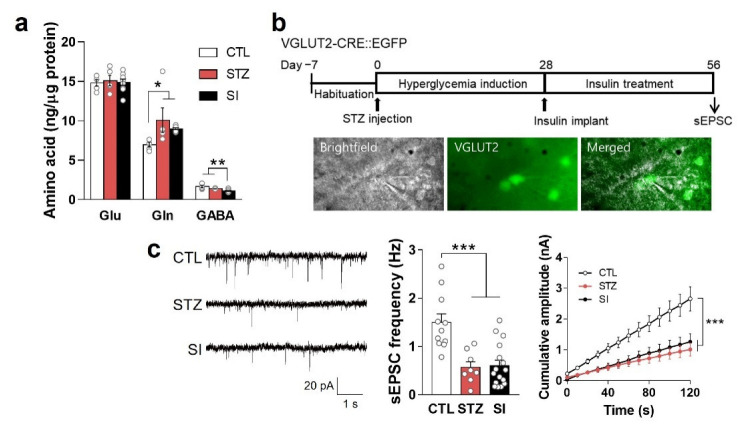
The amount of neurotransmitter and hypoactive glutamatergic signaling did not change with insulin treatment. (**a**) Glu, Gln, and GABA levels in the PFC were analyzed using LC-MS/MS. CTL, *n* = 5; STZ, *n* = 5; SI, *n* = 9. Data are presented as mean ± SEM. * *p* < 0.05, ** *p* < 0.01 between indicated groups according to one-way ANOVA with Newman–Keuls post-hoc tests. (**b**) Experimental design for electrophysiologic analysis and microscope images of sEPSC recording. sEPSC in the mPFC was measured after 4-week hyperglycemia induction and subsequent 4-week insulin treatment in VGLUT2-CRE::EGFP mice. (**c**) sEPSC frequency and cumulative amplitude in glutamatergic neurons in the mPFC. CTL, *n* = 11 cells from 2 mice; STZ, *n* = 8 cells from 2 mice; SI, *n* = 16 cells from 3 mice. Data are presented as mean ± SEM. *** *p* < 0.001 between indicated groups according to one-way ANOVA with Newman–Keuls post-hoc tests for sEPSC frequency and two-way ANOVA with Tukey’s post-hoc tests for cumulative amplitude.

We also investigated the effects of insulin treatment on Glu and Gln levels and found no significant differences between the STZ and SI groups (Figure 8a). Previously, we demonstrated that hypoactive glutamatergic signaling causes depressive behaviors [10,11,14]. Therefore, we hypothesized that the depressive-like behaviors of the SI group were due to hypoactive glutamatergic signaling in the mPFC, even when Glu and Gln levels were sufficient. Thus, we evaluated the sEPSC at 8 weeks in the STZ and SI groups. As expected, the STZ and SI groups exhibited a remarkably lower frequency and amplitude than the CTL group (Figure 8b,c), suggestive of hypoactive glutamatergic signaling in the mPFC, even when normoglycemia was achieved.

### 3.8. Hyperglycemia Decreased IRβ and Changed Its Localization in the mPFC

**Figure 9:**
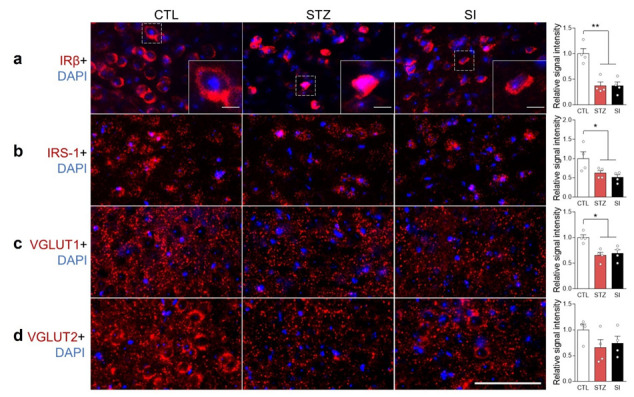
Hyperglycemia reduced insulin receptor β (IRβ) expression and altered its cellular localization in the mPFC. Immunohistochemistry images of (**a**) IRβ, (**b**) insulin receptor substrate-1 (IRS-1), (**c**) vesicular glutamate transporter 1 (VGLUT1), and (**d**) VGLUT2 with the nuclear marker DAPI in the mPFC. Scale bar = 50 μm. (**a**) Inset scale bar = 5 μm. The right panel shows each signal quantification result. *n* = 4/group. Data are presented as mean ± SEM. * *p* < 0.05, ** *p* < 0.01 between indicated groups according to one-way ANOVA with Newman–Keuls post-hoc tests.

**Figure 10:**
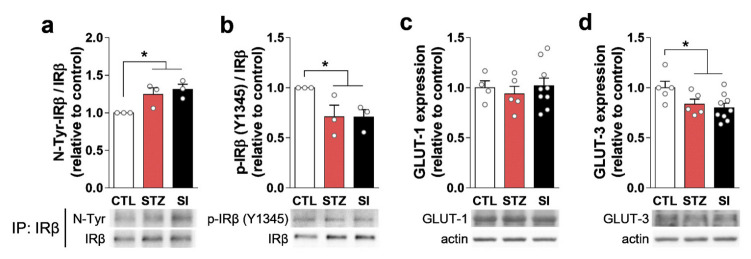
Hyperglycemia increased IRβ tyrosine nitration and decreased IRβ phosphorylation and glucose transporter (GLUT) expression. (**a**,**b**) Immunoprecipitation (IP) of IRβ and subsequent Western blot of (**a**) nitrotyrosine (N-Tyr) and (**b**) phospho-IRβ (Y1345). *n* = 3/group (three replicative IPs with pooled lysates). (**c**,**d**) Western blots of (**c**) GLUT-1 and (**d**) GLUT-3 in the PFC. CTL, *n* = 5; STZ, *n* = 5; SI, *n* = 9. Data are presented as mean ± SEM. * *p* < 0.05 between indicated groups according to one-way ANOVA with Newman–Keuls post-hoc tests.

Previous studies have suggested that insulin signaling could disrupt glutamate vesicular transport systems, affecting glutamate release [16,17]. We examined the expression and localization of IRβ, IRS-1, VGLUT1, and VGLUT2 in the mPFC. Insulin depletion decreased IRβ expression and dislocated IRβ from the membrane into the cytoplasm (Figure 9a). The expression of IRS-1 and VGLUT1 also decreased in the STZ group and did not recover with insulin treatment (Figure 9b–d). Interestingly, nitration of IRβ increased in the STZ group (Figure 10a), resulting in decreased phosphorylated IRβ in STZ-treated mice; this was not reversed with insulin (Figure 10b).

We also examined the expression of GLUTs in the PFC. GLUT-3 expression was decreased in the STZ group and did not recover with insulin treatment (Figure 10d). GLUT-1 expression did not change during hyperglycemia or glycemic control (Figure 10c).

## 4. Discussion

In this study, we showed that long-term STZ-induced hyperglycemia evoked depressive-like behaviors with long-lasting changes, and these depressive-like behaviors were likely the result of hypoactive glutamatergic neurotransmissions in the PFC. Additionally, we found long-lasting changes in IRβ, IRS-1, VGLUT1, SNAT2, PAG, and GLUT-3 in the PFC of animals treated with STZ. Our results suggest that further studies to determine how to reduce or reverse hyperglycemic-induced long-lasting changes in the PFC would be valuable to the development of new treatment strategies for the comorbidities of diabetes and major depressive disorder.

To investigate behavioral and physiologic changes associated with hyperglycemia and glycemic control, we utilized an STZ-induced hyperglycemia mouse model and insulin-pellet-implantation regime. In previous studies, rodents have usually been treated with insulin immediately or within 1 week after an STZ injection. As a result, these studies showed that insulin reversed depressive behaviors and hyperglycemia-induced damage in the brain [18,19]. By contrast, in this study, we maintained STZ-induced hyperglycemia for 4 weeks, which is equivalent to approximately 3 years in humans [20], to induce sufficient changes in the brain to mimic the difficult-to-recover brain damage of patients with diabetes. We then administered insulin over the same duration of time (4 weeks) to investigate whether prolonged insulin treatment reversed hyperglycemia-induced changes and to identify changes that were unresponsive to insulin treatment.

In our mouse model, prolonged hyperglycemia resulted in higher Gln levels, but no change in Glu, in the PFC (Figure 5b), which is consistent with the results of our previous study [6]. Glu in neurons is produced by PAG from Gln derived from astrocytes. In astrocytes, Gln is synthesized by GS from Glu, originating in the synaptic cleft and/or the tricarboxylic acid (TCA) cycle [21,22]. It is not surprising that Gln was increased in the PFC of the STZ and SI animals (Figure 5b and Figure 8a) because the expression and activity of GS were not decreased, and glucose was abundant (Figure 3b and Figure 7d). However, Glu was not increased in the STZ and SI groups (Figure 5b and Figure 8a), despite the fact that increased Gln could produce Glu. The expression of both SNAT2 and PAG was decreased by hyperglycemia and was not restored with insulin treatment (Figure 7g), which may have explained the lack of change in Glu in the STZ and SI groups compared with the CTL group (Figure 8a). Thus, prolonged hyperglycemia may increase Gln levels in astrocytes, but not Glu in glutamatergic neurons, because of a reduced number of PAG and Gln transporters.

In the present study, we also confirmed that STZ-induced hyperglycemia caused depressive-like behaviors with social interactive, anhedonic, and despair behavioral changes (Figure 1 and Figure 2). It is known that decreased activity of glutamatergic neurotransmission can lead to depressive and anhedonic behaviors [10,11]. However, despite the decrease in glutamatergic neurotransmission in the STZ mice, there was no change in GS activity, and the Gln level was increased in the PFC. These findings differ from the results of our previous studies in which GS activity and amounts of Glu and Gln in the PFC were decreased, resulting in hypoactive glutamatergic neurotransmission [10,11,14]. Thus, our current results suggest that factors other than GS activity and Glu–Gln levels have a greater influence on glutamatergic neurotransmission during long-term hyperglycemia.

In the brains of healthy adults, the Glu pool is tightly regulated by the Glu–Gln cycle to maintain adequate glutamatergic activity [23,24]. Although there was no difference in the availability of Glu in the PFC under hyperglycemic conditions, there was a decrease in VGLUT1 in glutamatergic neurons (Figure 9c). Moreover, both the amplitude and frequency of glutamatergic signaling were decreased (Figure 5e and Figure 8c). It is well established that a lesser number of Glu synaptic vesicles results in a lower amplitude of excitatory postsynaptic currents [17]. Therefore, a reduced number of Glu synaptic vesicles is the likely underlying cause of hypoactive glutamatergic neurotransmission during long-term hyperglycemia.

The expression of VGLUT1 and VGLUT2 is influenced by insulin signaling via insulin receptors [16,17]. In the present study, we found reduced IRβ and IRS-1 expression in the PFC of the STZ and SI group animals (Figure 9a,b). The subcellular localization of IRβ was not confined to the cell membrane, as some neurons exhibited cytoplasmic localization (Figure 9a). Moreover, nitrated tyrosine residues of IRβ were increased and the phosphorylated form of IRβ was decreased in the STZ and SI mice (Figure 10a,b). These results suggest that insulin signaling becomes abnormal in a prolonged hyperglycemic condition and does not recover with 4-week insulin treatment. Therefore, the possible dysfunction of insulin signaling in the PFC may result in a lower level of VGLUT1, which may lead to a reduced number of Glu synaptic vesicles.

The other possible explanation for hypoactive glutamatergic neurons is that neuronal Glu is preferentially used for ATP synthesis rather than synaptic transmission during prolonged hyperglycemia. As the brain is almost entirely dependent on glucose for its energy and neurotransmission needs, hyperglycemia could impact brain function directly [25]. GLUT-1 and GLUT-3 are involved in glucose delivery to astrocytes and neurons, respectively [26]. Previous studies suggest that both transporters are downregulated in chronic hyperglycemia [26,27]. In the present study, GLUT-3 expression was decreased in both the STZ and SI groups (Figure 10b). In this setting, neuronal Glu would be used as a substrate of the TCA cycle for energy production, instead of for synaptic neurotransmission [28].

Moreover, glutamatergic neurotransmission and neuronal/glial changes in the PFC were not restored to normal with insulin treatment. This finding implies that prolonged hyperglycemia (equivalent to approximately 3 years in humans) induces alterations in the brain, including damage to insulin receptors and the insulin signaling system in the PFC, which are not easily normalized by good blood glucose control. This can create a vicious cycle in which exogenous insulin has insufficient effects on the brain. Although the animal model we used did not perfectly mimic the long-term hyperglycemic situation in humans, our results also explain why depressive behaviors and neuronal activity changes in hyperglycemic mice, as well as depression symptoms in patients with diabetes, do not always improve when blood glucose is well controlled with insulin treatment [2]. These results emphasize the necessity of developing specialized treatments for depressive symptoms, in addition to blood glucose regulation, to improve the reduced glutamatergic neurotransmission caused by hyperglycemia. A strategy to restore insulin receptor damage is one option. RNS-induced tyrosine nitration of insulin signaling components impedes insulin signal transduction by competitively inhibiting tyrosine phosphorylation and activation, leading to insulin resistance [29,30]. Our results demonstrate that hyperglycemia-induced nitration and the low phosphorylation of tyrosine residues in IRβ are difficult to resolve with insulin treatment for a period equal to the duration of hyperglycemia. Therefore, elucidating the relationship between tyrosine nitration and insulin signaling dysfunction in type 1 diabetes is necessary, and controlling nitrative modification of the insulin signaling system could be the beginning of the development of drugs that improve depression caused by hyperglycemia.

To further solidify the results of this study, changes in neuronal activity and insulin signaling when insulin is treated for a longer period should also be investigated. We also plan to verify whether the activity of insulin receptors and the intrinsic activity of neurons are increased when a denitrative substance is treated. In addition, if it is demonstrated that the depressive behavior of hyperglycemic mice is improved when glutamatergic neurons are activated by methods such as optogenetics, our hypothesis will be supported.

## Data Availability

Not applicable.
